# Resveratrol induces apoptosis via ROS-triggered autophagy in human colon cancer cells

**DOI:** 10.3892/ijo.2012.1325

**Published:** 2012-01-03

**Authors:** HISANORI MIKI, NORIHISA UEHARA, AYAKO KIMURA, TOMO SASAKI, TAKASHI YURI, KATSUHIKO YOSHIZAWA, AIRO TSUBURA

**Affiliations:** Department of Pathology II, Kansai Medical University, Moriguchi, Osaka 570-8506, Japan

**Keywords:** resveratrol, reactive oxygen species, autophagy, apoptosis, caspase-8, colon cancer, HT-29, COLO 201

## Abstract

Resveratrol (Res; 3,4′,5-trihydroxy-*trans*-stilbene), which is a polyphenol found in grapes, can block cell proliferation and induce growth arrest and/or cell death in several types of cancer cells. However, the precise mechanisms by which Res exerts anticancer effects remain poorly understood. Res blocked both anchorage-dependent and -independent growth of HT-29 and COLO 201 human colon cancer cells in a dose- and time-dependent manner. Annexin V staining and Western blot analysis revealed that Res induced apoptosis accompanied by an increase in Caspase-8 and Caspase-3 cleavage. In HT-29 cells, Res caused autophagy as characterized by the appearance of autophagic vacuoles by electron microscopy and elevation of microtubule-associated protein 1 light chain 3 (LC3)-II by immunoblotting, which was associated with the punctuate pattern of LC3 detected by fluorescein microscopy. Inhibition of Res-induced autophagy by the autophagy inhibitor 3-methyladenine caused a significant decrease in apoptosis accompanied by decreased cleavage of Casapse-8 and Caspase-3, indicating that Res-induced autophagy was cytotoxic. However, inhibition of Res-induced apoptosis by the pan-caspase inhibitor Z-VAD(OMe)-FMK did not decrease autophagy but elevated LC3-II levels. Interestingly, Res increased the intracellular reactive oxygen species (ROS) level, which correlated to the induction of Casapse-8 and Caspase-3 cleavage and the elevation of LC3-II; treatment with ROS scavenger N-acetyl cysteine diminished this effect. Therefore, the effect of Res on the induction of apoptosis via autophagy is mediated through ROS in human colon cancer cells.

## Introduction

Colon cancer is the second most prevalent cancer and the third leading cause of cancer deaths worldwide, resulting in almost half a million deaths every year (1,2). Surgical resection remains the only curative treatment for colorectal cancer, but the outcome is not always satisfactory. Only 70% of colorectal cancers are resectable, and 75% of the resectable cancers are curable. Many patients require adjuvant chemotherapy (3). Approximately 70–90% of colon cancers seem to be associated with dietary habits; therefore, their is interest in dietary factors that can exert cancer chemopreventive/chemotherapeutic action against colon cancer cells (4).

Resveratrol (Res; 3,4′,5-trihydroxy-*trans*-stilbene) is a natural polyphenolic product and a phytoalexin produced by grapes, mulberries, and peanuts, and it is widely present in red wine and other constituents of the human diet. Res has various biological activities, including anti-inflammatory, antifungal, antimutagenic and anticancer effects (5). In this respect, Res may be a potential anticancer agent in the humans (6). Res is not overtly toxic to animals when administered at doses high enough to achieve a pharmacological effect (7), and is gaining acceptance as a potential antitumor agent because of its pleiotropic effects through many intracellular signaling pathways (6,8). Res inhibits the growth of cancer cell lines derived from various origins, and this effect was associated with cell-cycle arrest and the induction of apoptosis (9–12).

Autophagy is an evolutionarily conserved catabolic process of degrading damaged proteins and/or organelles and recycling the materials to maintain the quality of cellular components (13). During autophagy, autophagosomes are formed by elongation of double membrane-bound vesicles, and they sequester cytoplasmic constituents. Subsequently, autophagosomes fuse with lysosomes to form autolysosomes in which the incorporated organelles are degraded. Autophagy also plays a role in human diseases including cancer. Emerging evidence indicates that chemotherapeutic agents induce autophagy in various types of cancer cells (14). On one hand, autophagy exerts a cell protective role that allows cells to survive against cytotoxic agents. On the other hand, autophagy results in cell death termed autophagic cell death or type II cell death (15,16). Recently, Res was shown to induce autophagy in several cancer cell lines (17–20). However, the ability of Res to induce autophagy and the role of autophagy in the production of a cell death signal or a cell survival signal in human colon cancer cells are largely unknown.

Several studies indicate that reactive oxygen species (ROS) production may mediate apoptosis and/or autophagy induction in several types of cancer cells (21–23). The relationship between ROS, autophagy, and apoptosis induced by Res in human colon cancer cells is still undefined. In the present study, we demonstrated the Res-induced cytotoxic effect in human colon cancer cells. A possible molecular mechanism involved is Caspase-8/Caspase-3-dependent apoptosis via ROS-triggered autophagy.

## Materials and methods

### Cell culture

A human colon cancer cell line, HT-29, was kindly provided by Dr Y. Katakura (Faculty of Agriculture, Kyusyu University, Fukuoka, Japan), and COLO 201 was obtained from the Japanese Cancer Research Resource Bank. HT-29 and COLO 201 cells were grown in DMEM and RPMI-1640, respectively, supplemented with 10% fetal bovine serum, and maintained at 37°C in a humidified atmosphere containing 5% CO_2_.

### Cell proliferation

Proliferation of HT-29 and COLO 201 cells was determined by using the 3-(4, 5-dimethylthiazol-2-yl)-2, 5-diphenyltetrazolium bromide (MTT, Sigma, St. Louis, MO, USA) assay and the 2-(2-methoxy-4-nitophenyl)-3-(4-nitrophenyl)-5-(2, 4-disulfophenyl-2H-thetra-zolium, monosodium salt (WST-8, Wako Chemical, Osaka, Japan) assay, respectively. Cells were seeded into 96-well plates at a concentration of 5×10^3^ HT-29 cells/well and 2×10^3^ COLO 201 cells/well. After 24 h of incubation, the cells were treated with 5 different concentrations (25, 50, 75, 100 and 150 μM) of Res (Sigma). The stock solution of Res (200 mM) was prepared by using dimethyl sulfoxide (DMSO) as the solvent. The absorbance was read at 540 nm for MTT (HT-29 cells) and 450 nm for WST-8 (COLO 201) by using an iMark microplate reader (Bio-Rad, Hercules, CA, USA).

### Soft agar colony formation assay

A soft agar colony formation assay was performed as described previously (24). First, 0.5% agarose in growth medium was added to six-well plates and allowed to solidify. Then, 1×10^4^ cells were plated triplicate in 0.3% agarose and were added to each well. The cells were incubated at 37°C in a 5% CO_2_ atmosphere for 13 days. Fresh growth medium (0.5 ml/well) was added after 1 week of incubation. At the end of the incubation period, colonies were stained with 0.005% crystal violet for 1 h and photographed. Colonies were counted by using the Image J imaging software developed at NIH.

### Western blot analysis

Western blots were performed as described previously (24). The following primary antibodies were used: anti-poly(ADP-ribose) polymerase (PARP, polyclonal) antibody, anti-Caspase-8 (1C12) antibody, anti-cleaved Caspase-3 (Asp175) (5A1E) antibody, anti-Bcl-xL (54H6) antibody and anti-Bax (polyclonal) antibody (Cell Signaling Technology, Beverly, MA, USA); anti-microtubule-associated protein 1 light chain 3 (LC3, polyclonal) antibody (Abgent, San Diego, CA, USA); and HRP-conjugated anti-actin (polyclonal) antibody (Santa Cruz Biotechnology, Santa Cruz, CA, USA).

### Electron microscopy

The ultrastructure of HT-29 cells after 150 μM Res treatment was determined by electron microscopy. After treatment with DMSO or 150 μM Res for 24 h, the cells were collected by centrifugation, washed with PBS, fixed in ice-cold Karnovsky’s solution (4% paraformaldehyde, 5% glutaraldehyde and 30 mM cacodylate buffer) at 4°C for 1 h, washed with 0.1 M cacodylate buffer several times, and incubated at 4°C overnight. The cells were then post-fixed in 2% osmium tetroxide at 4°C for 1 h, resuspended in 1% sodium alginate, collected by centrifugation, and gelated by adding 1 M calcium chloride. The gelated cells were dehydrated through a graded series of ethanol (50–100%) and propylene oxide and then processed for epon embedding. Semi-thin sections were stained with toluidine blue, and representative areas were chosen for ultrathin sectioning. Ultrathin sections were stained with uranyl acetate and lead citrate and examined with a JEM-1011 electron microscope (JEOL, Tokyo, Japan).

### Immunofluorescence

Immunofluorescence detection for LC3 was performed as described previously (25). HT-29 cells were grown on a 35-mm glass-bottom dish (Matsunami Glass, Osaka, Japan), treated with DMSO or 150 μM Res for 24 h, washed with PBS, and fixed in 10% neutral buffered formalin for 30 min at room temperature. The cells were washed in Tris-buffer (TBS) and blocked in TBS containing 5% bovine serum albumin at room temperature for 30 min. Cells were subsequently incubated overnight with anti-LC3 antibody (Cell Signaling), washed several times and then incubated with secondary antibody (Alexa Fluor 488 anti-rabbit IgG; Molecular Probes, Eugene, OR, USA) for 1 h. The cells were then counterstained with 4′6-diamidino-2-phenylindole (Dojindo Laboratories, Kumamoto, Japan) for 5 min. A laser scanning microscope (LSM510-META, Zeiss, Jena, Germany) was used to collect images of the cells.

### Apoptosis analysis

Apoptosis was analyzed by flow cytometry with Annexin V (Becton Dickinson, Franklin Lakes, NJ, USA) and propidium iodide (PI). A total of 4×10^5^ cells were seeded in each 50-mm culture dish. Twenty-four hours later, 150 μM of Res with the autophagy inhibitor 3-methyladenine (3-MA, Sigma) or the pan-caspase inhibitor Z-VAD (OMe)-FMK (Z-VAD, Enzo Life Sciences, Plymouth Meeting, PA, USA) were added. The cells were trypsinized, washed in cold PBS, and resuspended in 1X binding buffer at a concentration of 1×10^6^ cells/ml. Annexin V and PI solution were added to the cell preparations and incubated for 15 min in the dark at room temperature. Binding buffer was then added to each tube, and the samples were analyzed by using a FACSCalibur flow cytometer (Becton Dickinson) and CellQuest software (Becton Dickinson). For each sample, 10,000 cells were analysed.

### Flow cytometric methods for determination of total ROS

For determination of intracellular ROS levels, cells were grown in 6-well plates and treated with 150 μM Res for 24 to 72 h with or without ROS scavenger N-acetyl cysteine (NAC, Sigma). Cells were incubated with 5 μm 5-(and-6)-chloromethyl-2′,7′-dichlorodihydro-fluorescein diacetate, acetyl ester (CM-DCHF-DA, Invitrogen, Carlsbad, CA, USA), which is cleaved by intracellular esterases and transformed into a fluorescent dye when oxidized at 37°C for 30 min. The samples were analyzed by using a FACSCalibur flow cytometer and CellQuest software. For each sample, 10,000 cells were analyzed.

### Statistical analysis

All discrete values, expressed as mean ± SD, were analyzed with the Student’s t-test. P-values less than 0.05 and 0.01 were considered to be significant and highly significant, respectively.

## Results

### Res inhibits anchorage-dependent and -independent growth of HT-29 and COLO 201 cells

**Figure 1:**
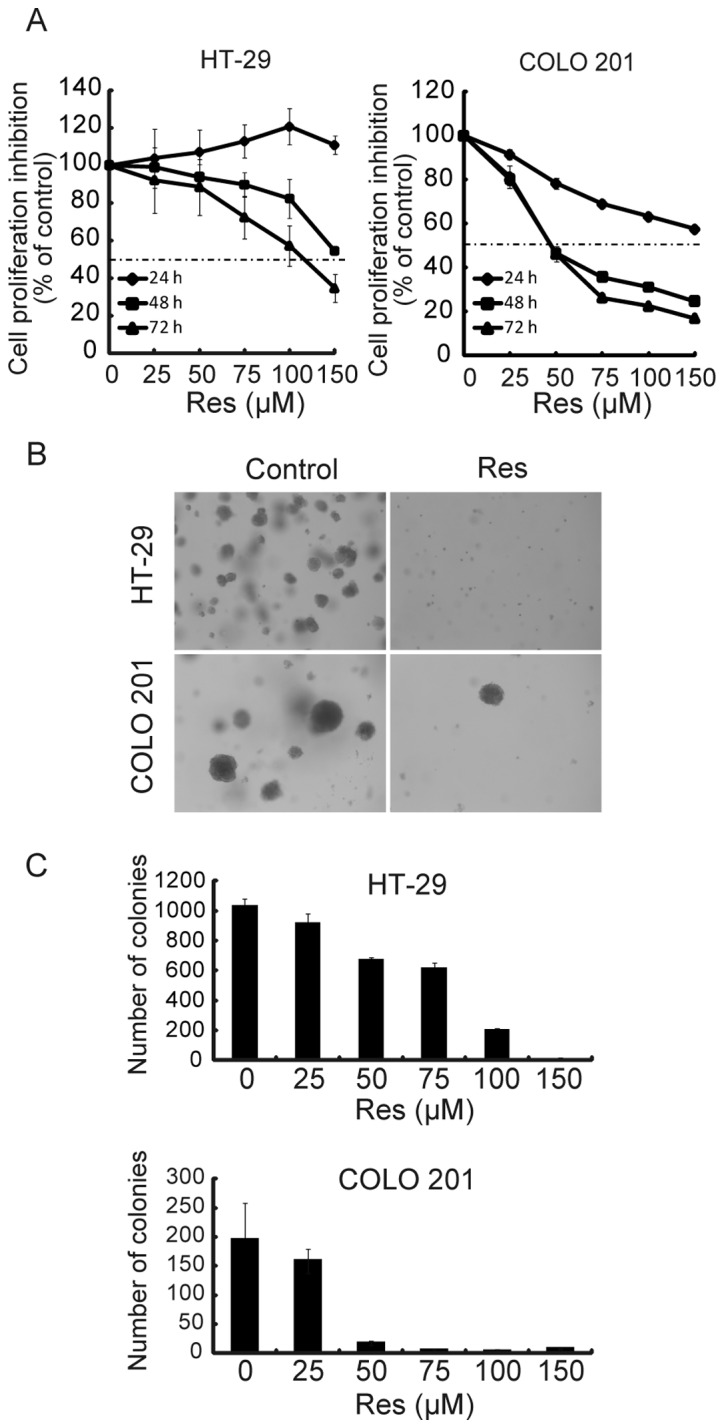
Effect of resveratrol on the cell proliferation of HT-29 and COLO 201 cells. (A) Dose- and time-dependent effect of Res on cell proliferation of HT-29 and COLO 201 cells. HT-29 and COLO 201 cells were plated on 96-well plates at 2×10^3^ cells/well and 5×10^3^ cells/well, respectively. HT-29 and COLO 201 cells were cultured with Res for the indicated times and at the indicated concentrations, and cell proliferation was determined by MTT and WST-8 assay, respectively. (B) Photomicrographs show representative colony formation from HT-29 and COLO 201 cells treated with Res. (C) Number of colonies after Res treatment in HT-29 and COLO 201 cells. Columns indicate mean ± SD.

To evaluate the effect of Res on cell proliferation, HT-29 and COLO 201 cells were treated with 5 different concentrations (25, 50, 75, 100 and 150 μM) of Res for up to 72 h. Res induced growth inhibition in a dose- and time-dependent manner (Fig. 1A). The half maximal inhibitory concentration (IC_50_) against HT-29 and COLO 201 cells after a 72-h treatment was 115.9 and 47.3 μM, respectively. To ascertain whether Res affected anchorage-independent growth, we assessed the ability of Res-treated cells to form colonies in soft agar. As shown in Fig. 1B and C, Res significantly reduced the number of colonies compared with the untreated cells in a dose-dependent manner. For the following studies, Res doses greater than the IC_50_ for 72 h were chosen (HT-29, 150 μM; COLO 201, 75 μM).

### Res induces apoptosis in HT-29 and COLO 201 cells

**Figure 2:**
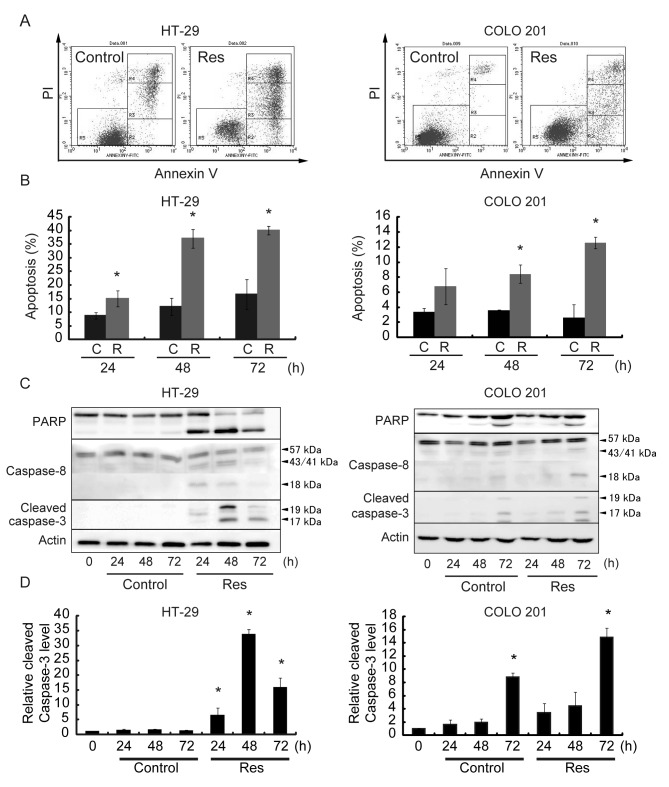
Resveratrol induces apoptosis in HT-29 and COLO 201 cells. HT-29 and COLO 201 cells were treated with 75 and 150 μM of Res, respectively, for 72 h. At each time point, apoptotic cells were identified with an Annexin V assay. (A) Representative histograms of Annexin V and PI staining of cells treated with Res for 72 h are shown. (B) Quantitative analysis of the percentage of apoptotic cells. ^*^p<0.05 vs. respective control cells. Columns indicate mean ± SD of three experiments. (C) Whole-cell lysates from control and Res-treated HT-29 and COLO 201 cells at the indicated time points were subjected to SDS-PAGE, and the levels of cleaved PARP, Caspase-8 and Caspase-3 were analyzed by immunoblotting. Actin was used as a loading control. (D) Blots were scanned and protein expression was quantified by densitometric analysis. ^*^p<0.05 vs. respective control cells. Columns indicate mean ± SD of three experiments.

An Annexin V assay showed that Res induced apoptotic cell death in a time-dependent manner. Representative results are shown in Fig. 2A, and quantitative data from 3 different experiments are summarized in Fig. 2B. The percentage of apoptotic cells was ~40% in HT-29 cells and ~12% in COLO 201 cells after a 72-h Res treatment. We then examined changes in the protein levels of apoptosis-related molecules. Representative results are shown in Fig. 2C, and quantitative data from 3 different experiments are summarized in Fig. 2D. Cells treated with Res exhibited an increase in the levels of PARP, Caspase-8 and cleaved Caspase-3. The levels of PARP, Caspase-8 and cleaved Caspase-3 peaked at 48 h after Res treatment in HT-29 cells and 72 h after Res treatment in COLO 201 cells. Res did not affect the levels of Bax or Bcl-xL in HT-29 and COLO 201 cells (data not shown). Thus, Res-induced apoptosis may be mediated through Caspase-8/Caspase-3 activation.

### Resveratrol induces autophagy in HT-29 and COLO 201 cells

**Figure 3:**
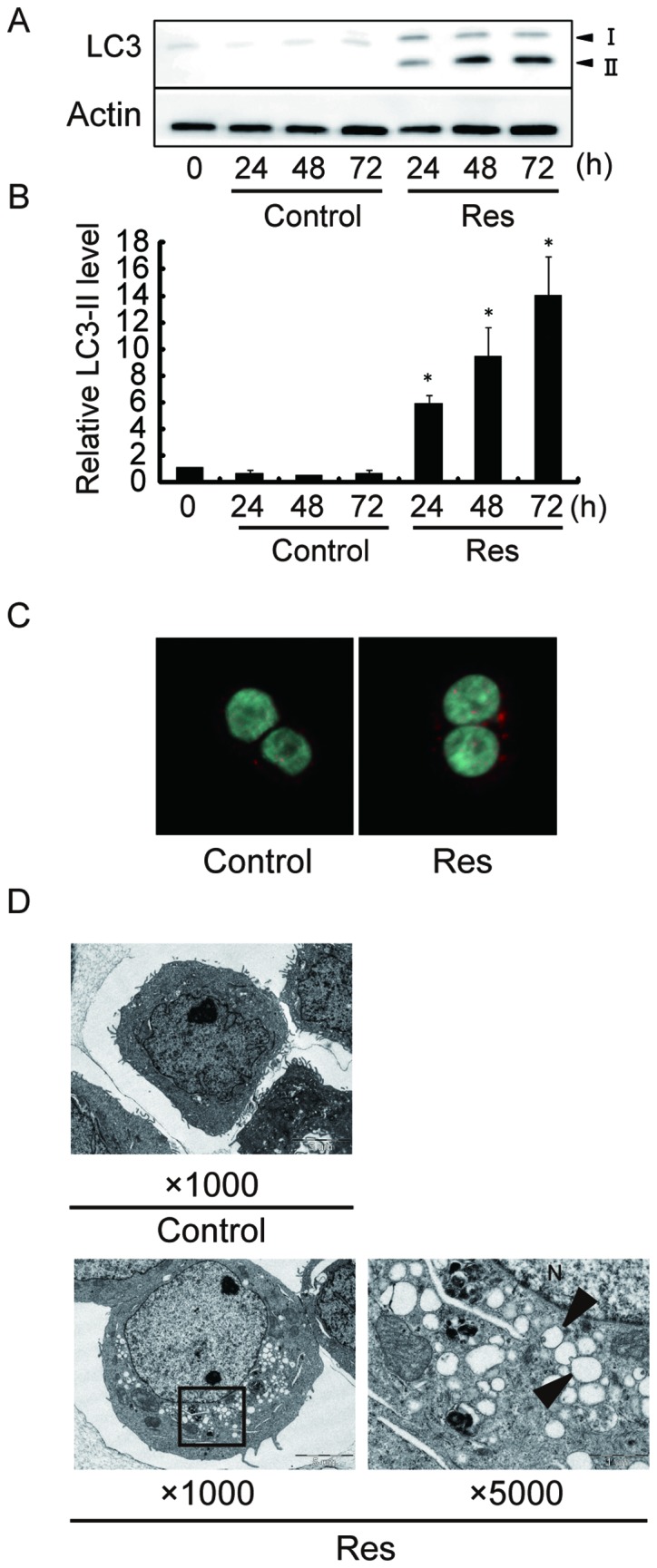
Resveratrol induces autophagy in HT-29 cells. (A) Whole-cell lysates from control and Res-treated HT-29 cells at indicated time points were subjected to SDS-PAGE, and the level of LC3 was analyzed by immunoblotting. Actin was used as a loading control. The time-dependent gradual increase in LC3-II levels peaked at 72 h after Res treatment. (B) Blots were scanned and protein levels were quantified by densitometric analysis. ^*^p<0.05 vs. respective control cells. Columns indicate mean ± SD of three experiments. (C) Detection of punctuate pattern of LC3 by confocal laser microscopy. Immunofluorescence image of DMSO control (left) and 150 μM Res-treated (right) HT-29 cells for 24 h. Note that Res-treated cells exhibited the punctuate pattern of LC3 (red). Cells were counterstained with 4′,6-diamidino-2-phenylindole (green). (D) Ultrastructural features were analyzed with electron microscopy after a 24-h treatment with Res. Numerous autophagosomes (arrowheads) were observed in Res-treated cells.

Next, we investigated the effect of Res on autophagy induction. Representative results are shown in Fig. 3A, and quantitative data from 3 different experiments are summarized in Fig. 3B. Western blot analysis revealed that the protein level of LC3-II increased by a maximum of ~15-fold in HT-29 cells after 72-h Res treatment as compared to control cells. The protein level of LC3-II increased by a maximum of ~3-fold in COLO 201 cells after 24-h Res treatment (data not shown) as compared to control cells. These data indicate that Res induced autophagy in HT-29 and COLO 201 cells. Although Res effectively suppressed the growth of both human colon cancer cell lines, HT-29 cells were chosen for the following detailed studies because the apoptotic as well as autophagic magnitude was much larger in HT-29 cells than in COLO 201 cells. We examined the localization of LC3 in HT-29 cells by immunofluorescence, and found punctuate patterns of LC3 fluorescence signals in Res-treated cells (Fig. 3C). By electron microscopy, numerous membranous vacuoles, autophagosomes containing residual materials, appeared in the cytoplasm of Res-treated cells, while there were relatively few such structures in the cytoplasm of control cells (Fig. 3D).

### Resveratrol induces autophagic cell death in HT-29 cells

**Figure 4:**
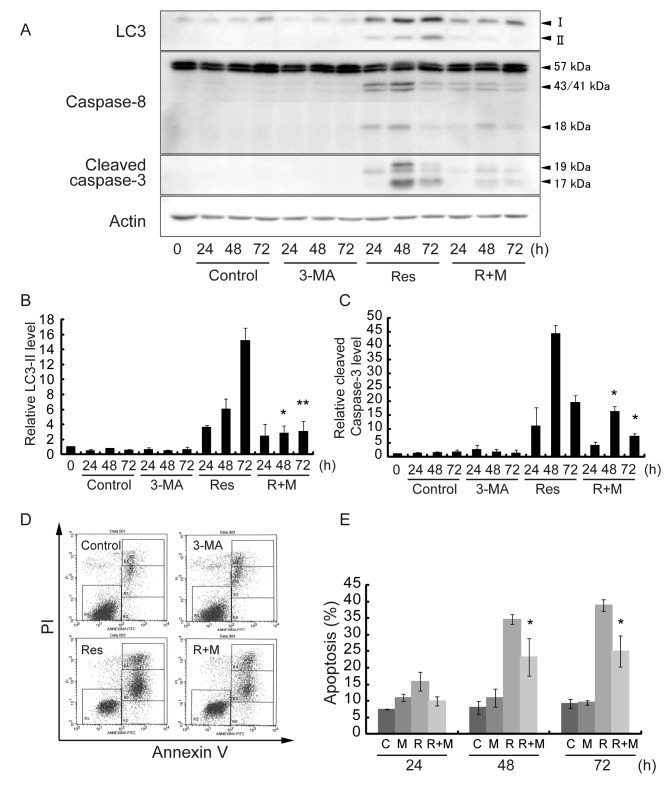
Effect of autophagy inhibitor 3-methyladenine on apoptosis in HT-29 cells. Untreated control cells (C) and cells treated with 3-MA (M), Res (R) and Res with 3-MA (R+M) for 72 h. (A) Representative data for LC3, Caspase-8 and cleaved Caspase-3 levels. (B and C) LC3-II and cleaved Caspase-3 levels were normalized to actin. The protein levels relative to the values for untreated control cells (normalized at 1) are shown. ^*^p<0.05 and ^**^p<0.01 vs. respective Res-treated cells. Columns indicate mean ± SD of three experiments. (D) Representative histograms of Annexin V and PI staining of cells treated with Res for 72 h are shown. (E) Quantitative analysis of the percentage of apoptotic cells by FACS analysis. Note the significant decrease in apoptosis in cells treated with Res in combination with 3-MA compared with Res treatment alone. ^*^p<0.05 vs. Res-treated cells. Columns indicate mean ± SD of three experiments.

To investigate the role of Res-induced autophagy in HT-29 cells, autophagy specific inhibitor 3-MA was used. Representative results are shown in Fig. 4A, and quantitative data from 3 different experiments are summarized in Fig. 4B and C. After 48- and 72-h Res treatment in the presence of 3-MA, the LC3-II level was significantly decreased as compared to Res treatment alone (Fig. 4A and B). The effect of 3-MA on cell viability and the induction of apoptosis was determined by Annexin V assay (Fig. 4D). Res treatment in the presence of 3-MA significantly reduced the percentage of apoptotic cells from 35 to 23% for a 48-h treatment and from 40 to 25% for a 72-h treatment as compared to Res treatment alone (Fig. 4E). We then examined the alterations in the protein levels of Caspase-8 and cleaved Caspase-3 after Res treatment and in the presence of 3-MA (Fig. 4A). The cleavage of Caspase-8 and Caspase-3 levels (Fig. 4C) were significantly deceased after Res treatment in the presence of 3-MA. These results indicate that Res induced autophagic cell death via the Caspase-8/Caspase-3 pathway in HT-29 cells.

### Z-VAD inhibits Res-induced cell death but not autophagy

**Figure 5:**
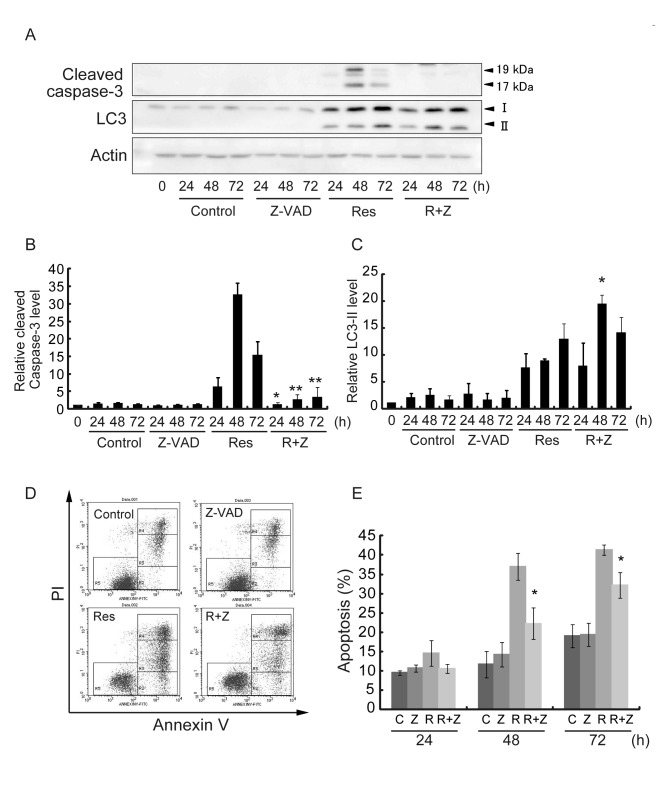
Effect of pan-caspase inhibitor Z-VAD (OMe)-FMK on autophagy in HT-29 cells. Untreated control cells (C) and cells treated with Z-VAD (Z), Res (R) and Res with Z-VAD (R+Z) for 72 h. (A) Representative data for the levels of cleaved Caspase-3 and LC3. (B and C) Cleaved Caspase-3 and LC3 levels were normalized to actin. The protein level relative to the value for untreated control cells (normalized at 1) is shown. These results indicate that Z-VAD inhibited Res-induced apoptosis but not autophagy. ^*^p<0.05 and ^**^p<0.01 vs. respective Res-treated cells. Columns indicate mean ± SD of three experiments. (D) Representative histograms of Annexin V and PI staining of cells treated with Res for 72 h are shown. (E) Quantitative analysis of the percentage of apoptotic cells by FACS analysis. ^*^p<0.05 vs. Res-treated cells. Columns indicate mean ± SD of three experiments.

To investigate the relationship between apoptosis and autophagy induced by Res in HT-29 cells, pan-caspase inhibitor Z-VAD was tested. Representative results are shown in Fig. 5A, and quantitative data from 3 different experiments are summarized in Fig. 5B and C. The cleaved Caspase-3 level after Res treatment in the presence of Z-VAD was significantly decreased at 24, 48 and 72 h after treatment, as compared with Res treatment alone (Fig. 5B). The effect of Z-VAD on cell viability and the induction of apoptosis were determined by Annexin V assay (Fig. 5D). Res treatment in the presence of Z-VAD reduced apoptotic cells from 35 to 23% for 48 h of treatment and from 40 to 32% for 72 h of treatment as compared to Res treatment alone at respective treatment periods (Fig. 5E). We then examined the alterations in the protein levels of LC3-II after Res treatment in the presence of Z-VAD. The protein level of LC3-II relative to the control cells significantly increased after Res treatment in the presence Z-VAD for 48 h after the treatment (10- vs. 20-fold) when Res-induced apoptosis peaked in HT-29 cells (Fig. 5C). These results indicate that Z-VAD inhibited Res-induced apoptosis but not autophagy and suggest that Res-induced autophagy may be located upstream of apoptosis.

### ROS mediates Res-induced autophagy and apoptosis

**Figure 6:**
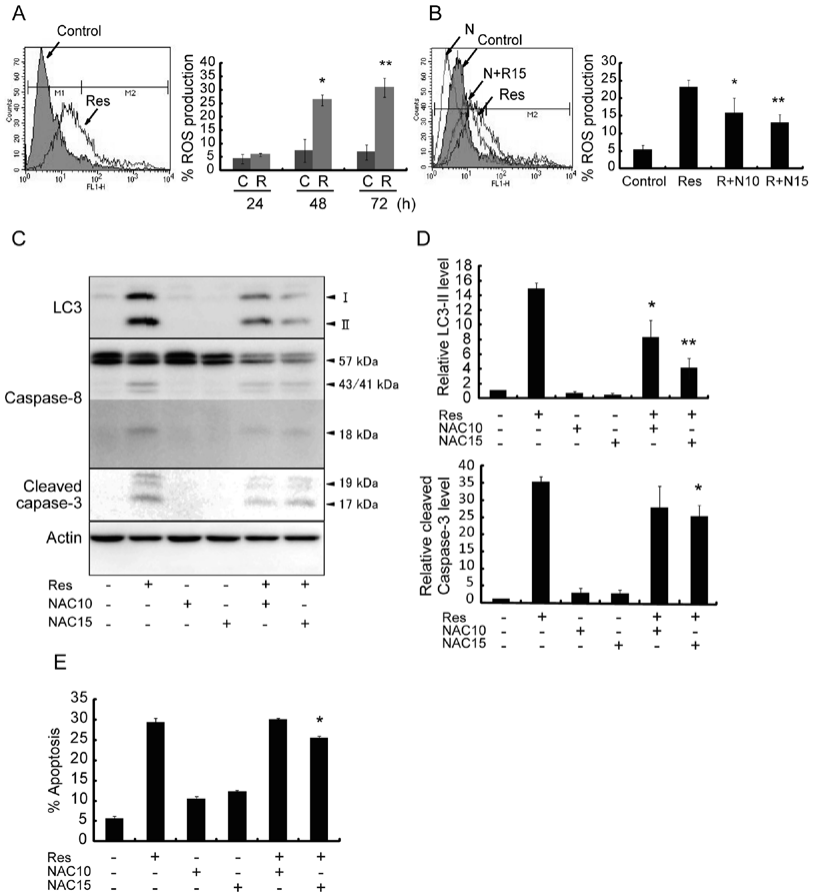
Intracellular ROS controls Res-induced autophagy and apoptosis. HT-29 cells were cultivated with DMSO or Res (150 μM) for 72 h, and ROS levels were determined by CM-DCHF-DA staining and flow cytometry. (A) Representative histograms of CM-DCHF-DA staining of cells treated with Res for 48 h are shown. Quantitative analysis of the percentage of ROS production determined by FACS analysis. Untreated control cells (C) and Res-treated cells (R). ^*^p<0.05 and ^**^p<0.01 vs. Res-treated cells. Columns indicate mean ± SD of three experiments. (B) Representative histograms of the CM-DCHF-DA staining of cells treated with Res and NAC for 48 h are shown. Quantitative analysis of the percentage of ROS production determined by FACS analysis. Untreated control cells (C), cells treated with Res (R), and cells treated with Res and NAC (10 mM, R+N10 and 15 mM, R+N15) for 48 h. ^*^p<0.05 and ^**^p<0.01 vs. Res-treated cells. Columns indicate mean ± SD of three experiments. (C) Representative data for LC3, Caspase-8 and cleaved Caspase-3 levels. (D) LC3 and cleaved Caspase-3 levels were normalized to actin. The protein levels relative to the values for untreated control cells (normalized at 1) are shown. ^*^p<0.05 and ^**^p<0.01 vs. Res-treated cells. Columns indicate mean ± SD of three experiments. (E) Quantitative analysis of the percentage of apoptotic cells as determined by Annexin V analysis. These data indicate that ROS controls autophagy and apoptosis in Res-treated HT-29 cells. ^*^p<0.05 vs. Res-treated cells. Columns indicate mean ± SD of three experiments.

We evaluated the effect of Res on the production of ROS and its involvement with apoptosis and/or autophagy. Res significantly increased intracellular ROS levels in a time-dependent manner (Fig. 6A). The percentage of ROS production was ~30% after Res treatment for 72 h, while incubation of cells with Res together with ROS scavenger NAC at a dose of 10 or 15 mM for 48 h significantly blocked the increase in ROS level from 23 to 15% and to 12%, respectively (Fig. 6B). At the same time, NAC decreased the Res-induced protein level of LC3-II relative to the control cells from 15-fold to ~8-fold and 4-fold, respectively (Fig. 6C and D). The protein levels of Caspase-8 and cleaved Caspase-3 were decreased, and the cleaved Caspase-3 level dropped from 35-fold to ~25-fold after cells were treated with Res in the presence of 15 mM NAC for 48 h (Fig. 6C and D), which was associated with a decrease in apoptosis (Fig. 6E). These data may indicate intracellular ROS as the upstream stimulus that controls both autophagy and apoptosis in Res-treated HT-29 cells.

## Discussion

We demonstrated that Res inhibited human colon cancer cell growth and found that Res induced Caspase-8/Caspase-3-dependent apoptosis through autophagy via ROS production. A concentration of 40 μM is relevant in terms of the possible biological effects of Res consumed from grape beverages (26). Thus, a Res dose of 150 μM for HT-29 cells and 75 μM for COLO 201 cells was relatively high, compared with physiological doses. However, here we demonstrated that exposure of HT-29 and COLO 201 cells to Res reduced cell proliferation rates in a dose- and time-dependent manner (Fig. 1). Induction of growth arrest and apoptosis is the central mechanism by which Res exerts antitumor effects against various types of cancers (9–12). When HT-29 and COLO 201 cells were exposed to 150 and 75 μM of Res, respectively, apoptosis was induced in a time-dependent manner, and the activity of apoptosis executor Caspase-3 was increased (Fig. 2). Then, the apoptosis cascade was examined. On one hand, Res was reported to induce apoptosis through the mitochondria pathway (27,28). On the other hand, the effect of Res involves Caspase-8/Caspase-3 signaling and induction of apoptosis via the death receptor pathway in several cancer cell lines (29–31). Our study showed that Res did not affect the Bax and Bcl-xL levels (data not shown), whereas the increased activity of Caspase-8/Caspase-3 (Fig. 2) indicates that Res-induced apoptosis may be mediated through the death-receptor pathway.

Res was found to induce autophagy in several cancer cells from different origin (17–20). In the present study, we found that Res induced autophagy in HT-29 and COLO 201 cells and that the magnitude of Res-induced apoptosis and autophagy was different in the different colon cancer cell lines (Figs. 2 and 3). ‘Autophagic cell death’ can be verified by experiments with an autophagy inhibitor, and it can be defined by characteristic cell morphology (32–34). We demonstrated that inhibition of autophagy by 3-MA significantly lowered Res-induced cytotoxicity by decreasing Caspase-8 and Capase-3 levels; thus, autophagy functioned as the cell death mechanism (Fig. 4). To determine whether autophagy and apoptosis may precede each other or co-occur, inhibition studies were performed. The inhibition of autophagy by 3-MA suppressed apoptosis. In contrast, inhibition of apoptosis by Z-VAD accelerated autophagy (LC3-II accumulation) (Fig. 5). Blocking autophagy significantly decreased the Caspase-8/Caspase-3 levels, whereas blocking apoptosis increased the LC3-II levels, suggesting that autophagy initiates apoptosis. There is evidence that LC3 mediates apoptosis via the Caspas-8/Caspase-3 pathway (35).

Not only apoptosis but also autophagy are mediated via ROS production (22,23). Res is an antioxidant (36), and antioxidants exert different biological activities in cancer cells and in non-transformed cells. Antioxidants including Res effectively induced apoptosis in HT-29 cells via increased ROS production (37–39). In the present study, Res treatment time-dependently increased ROS production, and the quenching of ROS by NAC abolished Res-induced autophagy (reduced LC3-II levels) and apoptosis (decreased Caspase-8/Caspase-3 levels). Therefore, Res caused apoptosis and autophagy via the production of ROS (Fig. 6).

In conclusion, Res effectively suppressed the growth of HT-29 and COLO 201 human colon cancer cells. The possible molecular mechanisms involved are Caspase-8/Caspase-3-dependent apoptosis via ROS-triggered autophagy. Res in combination with ROS- and autophagy-inducers may be a possible therapy for colon cancer control.
